# An Implementation Trial to Improve Tobacco Treatment for Cancer Patients: Patient Preferences, Treatment Acceptability and Effectiveness

**DOI:** 10.3390/ijerph17072280

**Published:** 2020-03-28

**Authors:** Jennifer H. LeLaurin, Jesse Dallery, Natalie L. Silver, Merry-Jennifer Markham, Ryan P. Theis, Deandra K. Chetram, Stephanie A. Staras, Matthew J. Gurka, Graham W. Warren, Ramzi G. Salloum

**Affiliations:** 1 Department of Health Outcomes and Biomedical Informatics, College of Medicine, University of Florida, Gainesville, FL 32610, USA; jlelaurin@ufl.edu (J.H.L.); rtheis@ufl.edu (R.P.T.); chetramd@ufl.edu (D.K.C.); sstaras@ufl.edu (S.A.S.); matthewgurka@ufl.edu (M.J.G.); 2 Department of Psychology, University of Florida, Gainesville, FL 32611, USA; dallery@ufl.edu; 3 Department of Otolaryngology, College of Medicine, University of Florida, Gainesville, FL 32610, USA; Natalie.Silver@ent.ufl.edu; 4 Division of Hematology/Oncology, Department of Medicine, College of Medicine, University of Florida, Gainesville, FL 32610, USA; merry.markham@medicine.ufl.edu; 5 Department of Cell and Molecular Pharmacology and Department of Radiation Oncology, Medical University of South Carolina, Charleston, SC 29425, USA; warrengw@musc.edu

**Keywords:** cancer, oncology, smoking cessation, tobacco dependence, implementation

## Abstract

Continued smoking after a cancer diagnosis increases mortality, risk of recurrence, and negatively impacts treatment effectiveness. However, utilization of tobacco use cessation treatment among cancer patients remains low. We conducted a clinical trial assessing patient preferences, treatment acceptability, and preliminary effectiveness (7-day point prevalence at 12 weeks) of three tobacco treatment options among cancer patients at an academic health center. Implementation strategies included electronic referral and offering the choice of three treatment options: referral to external services, including the quitline (PhoneQuit) and in-person group counseling (GroupQuit), or an internal service consisting of 6-week cognitive behavioral therapy delivered via smartphone video conferencing by a tobacco treatment specialist (SmartQuit). Of 545 eligible patients, 90 (16.5%) agreed to enroll. Of the enrolled patients, 39 (43.3%) chose PhoneQuit, 37 (41.1%) SmartQuit, and 14 (15.6%) GroupQuit. Of patients reached for 12-week follow-up (n = 35), 19 (54.3%) reported receiving tobacco treatment. Of all patients referred, 3 (7.7%) PhoneQuit, 2 (5.4%) SmartQuit, and 2 (14.3%) GroupQuit patients reported 7-day point prevalence abstinence from smoking at 12 weeks. Participants rated the SmartQuit intervention highly in terms of treatment acceptability. Results indicate that more intensive interventions may be needed for this population, and opportunities remain for improving reach and utilization.

## 1. Introduction

Upward of 17% of cancer survivors in the United States are current tobacco users [1]. Continued tobacco use among cancer survivors reduces the effectiveness of cancer treatments and is associated with increased overall and cancer-specific mortality, increased risk for recurrence and second primary cancers, and diminished quality of life after treatment [2,3,4,5,6,7,8]. Most cancer patients who attempt to quit smoking do so without assistance, reducing their chances of success [9,10]. Barriers to smoking cessation in cancer patients include depression and anxiety, pain, stigma, and high levels of addiction [11,12,13,14,15,16,17]. Cancer patients may benefit from novel smoking cessation interventions that are accessible and tailored to their treatment context [18]. Further, oncologists can play a key role in connecting patients to these interventions. 

Promoting tobacco cessation among cancer patients has been referred to as the “fourth pillar of cancer care”, along with surgery, radiotherapy, and chemotherapy [19]. Delivery of comprehensive tobacco treatment in oncology settings is effective [13,20], and the routine assessment of tobacco use and the provision of tobacco dependence treatment are recommended by all major professional and advocacy groups in oncology [21,22]. Guidelines issued by the National Comprehensive Cancer Network specify that all cancer patients should receive evidence-based counseling and pharmacotherapy, along with close follow-up and retreatment when necessary [22].

Despite these efforts, adherence to tobacco treatment guidelines is suboptimal. Survey data indicate that although most oncologists screen their patients for tobacco use advise them to quit, only 44% discuss medication options and 39% provide cessation treatment or referral their patients to cessation services [23]. Whereas assessment of tobacco use may be common in oncology settings, improved cessation outcomes will be realized only if patients are also offered counseling and tobacco treatment support [24]. Barriers to provision of tobacco treatment in cancer care include lack of provider and staff training, low provider self-efficacy, and perceived patient resistance [23,25].

Due to the competing demands of complex cancer care, cancer centers are challenged with implementing strategies that both assess tobacco use and link tobacco users with tobacco treatment services [26,27]. Tobacco treatment for cancer patients may be improved substantially with solutions to consistently identify tobacco use and provide patient-centered treatment, while minimizing the burden on clinical staff. Recommendations for promoting tobacco treatment in cancer care include improving the accessibility of tobacco treatment interventions through use of technology and connecting cancer patients with cessation resources during clinic visits [19]. However, the factors affecting successful implementation of these strategies in diverse contexts, along with optimal delivery methods for tobacco treatment interventions for cancer patients, remain unclear. 

This study attempts to address this knowledge gap by investigating implementation strategies designed to improve uptake of tobacco treatment in cancer care. Given the barriers to incorporating tobacco treatment in oncology care, we used strategies designed to complement existing clinical practices (i.e., brief provider counseling) and extend tobacco treatment beyond the clinical visit. We took a patient-centered approach by multiple tobacco treatment options. Treatment options included an in-house dedicated 6-week cognitive behavioral therapy (CBT) program delivered via mobile phone video teleconference (SmartQuit) in addition to referral to existing state-run evidence-based tobacco treatment programs—a quitline (PhoneQuit) and in-person group sessions (GroupQuit). The SmartQuit treatment options was provided in order to offer intensive tobacco use treatment that would be convenient and accessible for cancer patients. 

We conducted this pilot trial as part of a larger study investigating the feasibility of implementing multi-level strategies to improve the access and utilization of tobacco treatment services for cancer patients. The objective of this trial was to assess provider counseling practices, program reach, factors influencing cancer patients’ treatment choices, and treatment acceptability. As a secondary objective, we investigated preliminary effectiveness (7-day point prevalence at 12 weeks) of tobacco treatment among cancer patients. 

## 2. Materials and Methods

This implementation study consisted of two components: (1) provider and staff training on best practices, and (2) providing patients with multiple tobacco treatment options. All other components of standard care (screening, brief provider counseling, referral, and medication prescription) remained in place during study activities.

### 2.1. Provider and Staff Training

Prior to patient enrollment, the study team conducted a 30 min training session for clinic staff based on the American Society of Clinical Oncology’s Tobacco Cessation Guide for Oncology providers and staff [28]. The training covered the rationale and clinical best practices for addressing tobacco use in cancer patients. Clinic staff were also trained to use electronic health record (EHR) referral to tobacco cessation treament services.

### 2.2. Participants and Setting

The trial was conducted among cancer patients visiting outpatient clinics in the University of Florida Health System, a large academic health center in the Southeastern U.S. Patients were eligible if they were ≥18 years old, had a cancer diagnosis, and were current smokers (defined as at least one cigarette in the previous week). Patients who were not ready to quit but interested in reducing their smoking or obtaining more information about quitting were eligible. Informed consent was obtained for all participants. Participants who completed the follow-up telephone call at 12 weeks received a $20 gift card. This study was approved by the University of Florida Institutional Review Board and is registered with ClinicalTrials.gov under NCT03482583, 2018.

### 2.3. Recruitment

We recruited participants from three oncology clinics from May 2018 to July 2019. Potential participants were identified by generating a daily list of scheduled patients with a “current smoker” status listed in the EHR. Clinicians and staff additionally identified patients. After eligible patients were identified, the RA alerted clinical staff who then asked patients if they were willing to talk to an RA about a smoking cessation study. If the patients agreed, the RA explained the study in detail and assessed eligibility. Eligible patients could choose either the standard referral to free state-run cessation services (PhoneQuit or GroupQuit) or the additional SmartQuit option. Only participants with smartphones were eligible for SmartQuit. If patients were interested in cessation services but did not want to participate in the study, they were referred to the state-run cessation program.

### 2.4. Treatment

#### 2.4.1. External Tobacco Treatment Services (PhoneQuit, GroupQuit)

The state of Florida provides free tobacco cessation services through a Quitline (PhoneQuit) and in-person group sessions (GroupQuit). Individuals who participate in these programs receive counseling and are eligible to receive a free 2-week supply of nicotine replacement therapy (NRT). The programs offer information about other cessation medications, including prescription options. If participants are interested in prescription cessation medications, they are instructed to contact their physician. GroupQuit sessions are held in every Florida county and are offered as a one-time 90-min program or as a series of four to six weekly sessions lasting 60 min each. PhoneQuit consists of three 15 to 20 min sessions. Content and materials for both programs are evidence-based and developed by the program. For patients choosing GroupQuit or PhoneQuit who had not already been referred to the state-run program, the RA provided a referral request form to the patient’s clinician or nurse clinician or nurse, who then entered a referral to the state-run tobacco cessation program the EHR system. Once the EHR referral was placed, the patient received a proactive telephone call from the service staff to complete enrollment.

#### 2.4.2. SmartQuit

We developed the evidence-based SmartQuit intervention to leverage the convenience and growing ubiquity of smartphone technology to deliver CBT and verify abstinence [29]. SmartQuit is similar to PhoneQuit in terms of convenience, but provides more intensive support and is tailored to cancer patients. The SmartQuit condition consisted of 6 weekly 30-min sessions with a certified tobacco treatment specialist (TTS) conducted via videoconferencing. Prior to the study, each TTS received intensive training on tobacco treatment best practices from a program accredited by the Council for Tobacco Treatment Training Programs. Additionally, they received specific training on treating cancer patients for tobacco dependence. SmartQuit sessions were scheduled in advance in accordance with patient preferences. The TTS followed a treatment manual that was based on the National Cancer Institute’s “Clearing the Air” curriculum, which covers topics such as addiction, withdrawal, dealing with triggers, overcoming cravings, and relapse prevention. The sessions were additionally tailored to included discussion of issues specific to cancer patients (e.g., impact of smoking on treatment outcomes). Consistent with the state-run cessation services, SmartQuit participants received a 2-week supply of NRT patches upon enrollment. During SmartQuit sessions, the TTS discussed other cessation medication options with the participants and instructed them to contact their physician if they were interested in prescription medications. 

For those choosing SmartQuit, the RA assisted with download of Vidyo^®^, a HIPAA-compliant video conferencing application, on the patient’s smartphone. The RA also provided the patients with a smartphone-compatible CO monitor (iCO, coVita, Santa Barbara, CA, USA), assisted them with downloading the accompanying Smokerlyzer© mobile application, and trained the patient on their use. The patients were told that the CO monitor would be used to verify smoking abstinence but they were free to use it for personal CO tracking if desired.

### 2.5. Measures

#### 2.5.1. Patient Exit Interviews (PEIs)

To assess provider adherence to tobacco treatment guidelines, the RAs administered PEIs after recruitment with all participants (regardless of tobacco treatment choice) in REDCap via a tablet. The PEI is a brief patient-reported assessment of provider delivery of counseling [30]. A PEI index score (0–10) is the sum of intervention steps each patient reports that the provider implemented. Tobacco use and socio-demographics (e.g., age, gender, race/ethnicity, rural/urban residence) were obtained using questions from the PhenX toolkit [31]. The PEI was administered at baseline collected reasons for tobacco treatment selection, past-7-day point prevalence of cigarette smoking, number of cigarettes per day, cigarette type, age of smoking onset, and cancer site and time since diagnosis. Reasons for tobacco treatment choice included binary response options (yes/no) and participants could select multiple responses. 

#### 2.5.2. Follow-Up Telephone Survey

A telephone survey was administered by a trained research staff member at week 12, regardless of group. The survey assessed treatment acceptability and smoking outcomes. Up to three contact attempts were made for each participant.

*Treatment Acceptability Questionnaire (TAQ):* The TAQ consisted of 16 items scored on a 0 to 10 scale (0 = worst, 10 = best). The questions assessed aspects of treatment acceptability, such as helpfulness in reducing smoking and thoughts and feelings related to the treatment. Questions also related to barriers and facilitators of tobacco treatment, including open-ended questions assessing suggestions for improvement. 

*Smoking Status:* We collected self-reported prolonged abstinence (i.e., sustained abstinence after an initial period in which smoking is not counted as a failure) and past-7-day point prevalence of cigarette smoking [32]. The TTS observed SmartQuit participants via video conferencing while they used the CO monitor to confirm smoking abstinence. CO readings were not obtained from GroupQuit or PhoneQuit participants because many did not own smartphones. Abstinence was defined as CO sample ≤4 ppm and reporting not smoking in the last 7 days [33].

### 2.6. Statistical Analysis

SPSS 25 (IBM, Armonk, NY, USA) was used to conduct statistical analysis [34]. Descriptive statistics were used to summarize baseline and outcome data. As an implementation outcome, reach was defined as the number of smokers referred to any tobacco treatment divided by the total number of smokers visiting the clinics over the study period, which was obtained via EHR report. We calculated rates of treatment option choice and utilization for each treatment option. For SmartQuit participants, treatment initiation was defined as completing at least one session and treatment completion as completion of all six sessions. For GroupQuit and PhoneQuit participants, treatment utilization was defined as participation in at least one session; this was assessed via self-report because we did not have access to the state-run program data. For secondary outcomes, we measured self-reported prolonged abstinence at week 12 and the change (baseline to week 12) in past-7-day smoking point prevalence. Abstinence outcomes were calculated using both complete case analysis (CCA) and a modified intent-to-treat (ITT) approach in which all patients (regardless of treatment utilization) are included in analysis and patients who were not reached for follow-up are considered smokers. 

## 3. Results

### 3.1. Participants

A total of 90 participants enrolled in the study and 35 (38.9%) completed the follow up survey. Most participants were non-Hispanic white and approximately one-third resided in rural areas. About half of the participants received a cancer diagnosis in the previous six months and the majority (65.6%) had diagnoses of head and neck or lung cancer. Baseline participant characteristics are presented in Table 1.

### 3.2. Reach and Tobacco Treatment Choice

A total of 545 cancer patients receiving care in the clinics during the study period were identified in the EHR as smokers. Of these, we enrolled 90 (16.5%) patients in the study. All GroupQuit and PhoneQuit enrollees received an electronic referral to external cessation services and study staff made at least three attempts to schedule initial sessions with the SmartQuit participants. Rates of enrollment differed among clinics, with the largest proportion of patients reached in the Medical Oncology clinic (see Table 2). 

Reasons for tobacco treatment choice are presented in Table 3. For GroupQuit, perceived treatment effectiveness and treatment-specific characteristics (i.e., having in-person group support) were most frequently cited. Over half of PhoneQuit and SmartQuit participants said they chose their treatment because it was convenient and they could complete it from home.

### 3.3. Smoking Outcomes

Smoking outcomes are presented in Table 4. Using intent-to-treat analysis, seven of 90 (7.8%) of participants were abstinent at 12 weeks. Among follow-up completers only, seven of 35 (20.6%) were abstinent at 12 weeks; of these, five received tobacco treatment. Abstinence rates were highest for the patients using GroupQuit and reduction in cigarettes per day was greatest among patients using quitline.

### 3.4. Treatment Use and Acceptability

About half of the patients completing follow-up received some form of tobacco treatment program (see Table 4). Among patients who selected SmartQuit, seven (18.9%) initiated treatment and five (13.5%) completed all treatment sessions. TAQ responses indicated high satisfaction with the SmartQuit program; patients rated the program highly in terms of ease of use and flexibility, and all gave the highest rating when asked if they would recommend the program to others (see Table 5).

## 4. Discussion

The current study reports on an implementation trial designed to promote uptake of evidence-based tobacco treatment in oncology clinics. We offered patients a choice of three treatment options and explored motivations for patients’ treatment choice as well as the acceptability of each treatment option. We additionally explored smoking cessation outcomes in our study sample. 

To better understand patient preferences for tobacco treatment, we used a pragmatic design and offered patients multiple treatment options rather than using random assignment. Each treatment option had unique features which appealed to different patients; GroupQuit offered face-to-face group support, PhoneQuit offered brief support that could be obtained from home, and SmartQuit offered more intensive support and could also be completed at home. The variation in treatment selection and reasons for choice suggest that multiple treatment options may enhance patient engagement. Treatment choice was most often motivated by convenience and perceived effectiveness of the treatment. Notably, for patients choosing SmartQuit and PhoneQuit, convenience was more frequently endorsed as a reason for treatment choice than the expectation that the treatment would be effective. Convenience may have been especially important in this study as nearly one-third of participants resided in rural areas. These findings should be interpreted in light of the fact that patients without smartphones were not able to participate in the SmartQuit intervention.

Participants reported relatively high provider adherence to screening and counseling best practices, consistent with previous literature [23,27]. However, screening and brief provider counseling is insufficient to fully address tobacco use in cancer patients—patients must be connected with cessation treatment. Our implementation strategies resulted in 16.5% of current smokers being enrolled in the study and referred to tobacco cessation resources. While we sought to have an RA present in each clinic at all times, this was not always achieved due to resource limitations; thus, the assessment of reach of the intervention may be underestimated. On the other hand, we may have missed patients who were referred to tobacco treatment services but did not enroll in the study. Nonetheless, the reach of this trial was within the range achieved by other programs designed to integrate tobacco treatment into cancer care. For example, programs participating in the Cancer Center Cessation Initiative (C3I), a National Cancer Institute-funded effort to implement evidence-based tobacco treatment into cancer care, have shown median reach of 17.1% [25]. Our finding that reach varied significantly by clinic (10.9–23.4%) indicates the need for implementation strategies tailored to individual clinic workflows and patient characteristics. 

While the SmartQuit treatment option was only provided in the context of this research study, the electronic referral to state-run cessation services (PhoneQuit, GroupQuit) remains part of standard care for all patients in this study’s health system. As oncology providers typically deliver tobacco screening and counseling, but do not routinely provide cessation treatment [23,27], the implementation of the electronic referral is an important step in ensuring the sustainability of tobacco treatment programs. With the ever-increasing demands on providers and clinic staff, the challenge lies in developing strategies to ensure that such referrals are consistently used with all patients. A possible strategy to increase the reach of tobacco treatment for cancer patients may be an automatic EHR referral of all cancer patients who smoke to these cessation services, thereby lessening the burden on providers [24]. However, cancer patients face significant barriers to cessation and may require more intensive efforts, such as warm hand-offs and in-person counseling. Finding the right balance between efficiency and effectiveness remains a challenge.

Provider counseling and referral is an important first step in addressing tobacco use in cancer patients, but treatment utilization is necessary in order to achieve cessation for most patients. About half of patients reached for follow-up completed some form of tobacco cessation treatment, which is higher than treatment utilization rates resulting from primary care referral to cessation programs [35,36,37]. However, the low rate of follow-up completion may yield a biased treatment result as we could not obtain treatment utilization data from the majority of our initial sample. Efforts to improve the accessibility and acceptability of tobacco treatment options for cancer patients may improve treatment utilization in this population. 

Using a modified intent-to-treat approach (i.e., non-respondents considered smokers) yielded lower abstinence rates than found in a meta-analysis of tobacco treatment interventions for cancer patients [38]. One explanation for this finding may be that the tobacco treatment options in this trial provided only a limited supply of NRT. Cancer patients who continue to smoke tend to be highly addicted to nicotine and often require prescription medications and combination therapy to achieve cessation [10,13,14]. Therefore, interventions to promote smoking cessation in cancer patients need to facilitate access to prescription medications in order to achieve maximum effectiveness. Interestingly, two patients enrolled in the study quit without utilizing a formal tobacco treatment program, although one of these participants had received NRT as part of the SmartQuit intervention. This might suggest that for some patients, a cancer diagnosis coupled with provider counseling may be sufficient intervention; however, for patients continuing to use tobacco well after a cancer diagnosis, more intensive interventions are likely needed.

Our smartphone-based intervention received high treatment acceptability ratings from patients. As expected, the technology-based SmartQuit program was rated highly for convenience and flexibility. The majority of cancer patients are older adults, representing the age group least likely to use the Internet or own smartphones; however, technology use in this population is rapidly increasing. From 2011 to 2016, smartphone use among adults over 65 rose from 11% to 42% [39]. A recent study found that over half of cancer patients surveyed would be willing to use smartphone technology for health monitoring and promotion [40]. Given the increasing utilization and acceptability of technology-based health interventions, smartphones are a promising delivery method for not only tobacco treatment but other health interventions as well.

A limitation of this study is the low proportion of patients who completed follow-up data collection, which may not have provided a representative sample and limited our ability to measure the effect of cessation treatment on patient outcomes. Further, our study only included patients who expressed interest in smoking cessation. We also did not evaluate the reasons patients refused treatment; this information could provide insight on how to optimize the timing and design of tobacco treatment interventions. While the prescription of cessation medication is a component of usual care in the participating clinics, another limitation of the research is that we did not collect data on cessation medication use or take additional steps to ensure that prescription cessation medication was prescribed to patients. Additionally, we did not conduct treatment fidelity checks for the SmartQuit sessions. Finally, participating clinics were not fully staffed with research assistants at all times, so the potential reach of the implementation strategies used may be underestimated. 

## 5. Conclusions

Provision of evidence-based tobacco cessation treatment is a critical step to improving cancer treatment outcomes. This study demonstrates the feasibility of using implementation strategies to incorporate tobacco treatment into routine oncology care; however, we demonstrate that there is much room for improvement in recruitment, follow-up, and treatment effectiveness. It is clear that more intensive efforts are required to successfully address tobacco use in oncology settings, which will require dedicated funding and resources. Implementation of tobacco treatment in oncology settings may benefit from a combination of more intensive approaches (e.g., warm hand-offs, on-site counseling) and strategies that reach a larger number of patients (e.g., automatic referrals). Future work should focus on developing multi-level strategies to improve reach, patient engagement, and effectiveness.

## Figures and Tables

**Table 1:** Participant characteristics (n = 90).

Characteristics	GroupQuit (n = 14)	PhoneQuit (n = 39)	SmartQuit (n = 37)	Total (n = 90)
Age (mean, SD)	57.3 (6.9)	60.6 (1.2)	55.5 (13.1)	58.0 (10.2)
Gender				
Male	4 (28.6%)	20 (51.3%)	16 (43.2%)	40 (44.4%)
Female	10 (71.4%)	19 (48.7%)	21 (56.8%)	50 (55.5%)
Race^2^				
White	9 (64.3%)	30 (76.9%)	29 (78.4%)	68 (75.6%)
Black/African American	4 (28.6%)	8 (20.5%)	5 (13.5%)	17 (18.9%)
Other	3 (17.0%)	2 (5.5%)	4 (10.8%)	9 (10.0%)
Ethnicity				
Hispanic	1 (7.1%)	0 (0.0%)	1 (2.7%)	2 (2.2%)
Non-Hispanic	13 (92.9%)	39 (100.0%)	36 (97.3%)	88 (97.8%)
Marital status				
Married or cohabitating	6 (42.9%)	24 (61.5%)	15 (40.5%)	45 (50.0%)
Not married or cohabitating	8 (57.1%)	15 (39.5%)	22 (59.4%)	45 (50.0%)
Rurality ^a^				
Urban	8 (57.1%)	28 (71.8%)	26 (70.3%)	62 (68.9%)
Rural	6 (42.9%)	11 (28.2%)	11 (29.7%)	28 (31.1%)
Cigarette smoking frequency				
Every day	13 (92.9%)	30 (76.9%)	34 (91.9%)	77 (85.6%)
Some days	0 (0.0%)	7 (17.9%)	3 (8.1%)	10 (11.1%)
E-cigarette use frequency				
Every day	1 (7.1%)	1 (2.6%)	4 (10.8%)	6 (6.7%)
Some days	1 (7.1%)	10 (25.6%)	6 (16.2%)	17 (18.9%)
Not at all	11 (78.6%)	27 (69.2%)	27 (73.0%)	65 (72.2%)
Cigarettes/day (mean, SD)	20.1 (15.1)	12.3 (8.5)	13.8 (12.1)	14.2 (11.5)
Age of smoking initiation (mean, SD)	21.0 (8.1)	18.9 (6.5)	16.2 (6.4)	17.0 (8.0)
PEI composite score (1–10)	6.9 (2.9)	7.2 (2.9)	7.7 (2.6)	7.4 (2.7)
Cancer diagnosis				
Past week	1 (7.1%)	2 (5.1%)	2 (5.4%)	5 (5.6%)
Past month	0 (0.0%)	4 (10.3%)	5 (13.5%)	9 (10.0%)
Past 1–3 months	3 (21.4%)	7 (17.9%)	4 (10.8%)	14 (15.6%)
Past 4–6 months	3 (21.4%)	7 (17.9%)	7 (18.9%)	17 (18.9%)
Past 7–12 months	2 (14.3%)	2 (5.1%)	3 (8.1%)	7 (7.8%)
Over 12 months	5 (35.7%)	17 (43.6%)	16 (43.2%)	38 (42.2%)
Cancer site ^b^				
Lung	4 (28.6%)	11 (28.2%)	11 (29.7%)	26 (28.9%)
Head and neck	5 (35.7%)	8 (20.5%)	20 (54.1%)	33 (36.7%)
Breast	0 (0.0%)	6 (15.4%)	7 (18.9%)	13 (14.4%)
Gastrointestinal	5 (35.7%)	11 (28.2%)	5 (13.5%)	21 (23.3%)
Thyroid	0 (0.0%)	2 (5.1%)	0 (0.0%)	2 (2.2%)
Genitourinary	2 (14.3%)	7 (17.9%)	3 (8.1%)	12 (13.3%)
Gynecologic	2 (14.3%)	1 (2.6%)	2 (5.4%)	5 (5.5%)
Bone	2 (14.3%)	4 (10.3%)	3 (8.1%)	9 (10.0%)
Brain	2 (14.3%)	4 (10.3%)	1 (2.7%)	7 (7.8%)
Hematologic/Blood	4 (28.6%)	3 (7.7%)	4 (10.8%)	11 (12.2%)
Other	1 (7.1%)	3 (7.7%)	1 (2.7%)	5 (5.6%)

^a^ Could choose more than response; ^b^ RUCA 1–3 = urban, RUCA 4–10 = rural.

**Table 2:** Reach.

Clinic	Eligible Patients	Enrolled Patients
Medical Oncology	227	53 (23.4%)
Radiation Oncology	230	25 (10.9%)
ENT	88	12 (13.6%)
Total	545	90 (16.5%)

**Table 3:** Reasons for tobacco treatment choice.

Reason for Treatment Choice	GroupQuit (n = 14)	PhoneQuit (n = 39)	SmartQuit (n = 37)
I think it would help me to quit	10 (71.4%)	18 (46.2%)	17 (45.9%)
I tried another method and it didn’t work out	2 (14.3%)	1 (2.6%)	2 (5.4%)
I know someone who is doing/has done the program	1 (7.1%)	0 (0.0%)	1 (2.7%)
I don’t have a smartphone	3 (21.4%)	7 (17.9%)	--
I would like having the group support	8 (57.1%)	--	--
I would rather talk to someone in-person	8 (57.1%)	--	--
It would be more convenient for me	--	26 (66.7%)	21 (56.8%)
I would like having the one-on-one support	--	8 (20.5%)	13 (35.1%)
I would rather talk to someone on the phone	--	15 (38.5%)	15 (40.5%)
I would like the fact that I can do it from home	--	26 (66.7%)	21 (56.8%)
I would like the video interaction with the counselor	--	--	8 (21.6%)
Other	2 (14.3%)	1 (2.6%)	3 (8.1%)

**Table 4:** Smoking outcomes at 12 weeks among follow-up completers (n = 35).

	GroupQuit (n = 5)	PhoneQuit (n = 18)	SmartQuit (n = 12)	Total (n = 35)
Treatment utilization				
Received treatment	3 (60.0%)	9 (50.0%)	7 (58.3%)	19 (54.3%)
No tobacco treatment	2 (40.0%)	9 (50.0%)	5 (41.7%)	16 (45.7%)
7-day abstinence				
Among patients completing follow-up (CCA) (n = 35)	2 (40.0%)	3 (16.7%)	2 (28.6%)	7 (20.0%)
Received treatment	2 (40.0%)	2 (11.1%)	1 (8.3%)	5 (14.3%)
No treatment	0 (0.0%)	1 (5.6%)	1 (8.3%)	2 (5.7%)
Among all patients (ITT) (n = 90) ^a^	2 (14.3%)	3 (7.7%)	2 (5.4%)	7 (7.8%)
Mean change in cigarettes/day (SD)	−12.3 (7.5)	−5.0 (6.3)	−4.7 (13.7)	−5.9 (8.9)

^a^ Non-respondents considered to be smokers.

**Table 5:** Treatment Acceptability Questionnaire (n = 17).

	GroupQuit (n = 2) M (SD)	PhoneQuit (n = 11) M (SD)	SmartQuit (n = 4) M (SD)
1. How interesting was the tobacco cessation treatment that you received?	8 (1.4)	4.0 (2.6)	9.25 (1.5)
2. How useful was the treatment that you received from this program?	4.5 (3.5)	6.2 (2.6)	8.75 (2.5)
3. How much new information did you learn as a result of the treatment that you received from this program?	3.5 (4.6)	4.7 (2.6)	8.5 (3.0)
4. How easy to understand was the treatment that you received from this program?	5.0 (7.0)	8.8 (2.0)	8.6 (2.5)
5. How satisfied were you with the treatment you received from this program?	8.5 (2.1)	6.7 (2.3)	10.0 (0.0)
6. How did this treatment compare with other treatments you have had for smoking cessation in the past?	0 (0.0)	5.0 (4.1)	8.6 (2.5)
7. How easily available was this program to you compared to other treatments you have had for smoking cessation in the past?	3.5 (5.0)	7.0 (4.0)	8.5 (2.4)
8. To what extent did the content of this treatment meet your specific needs compared to other treatments you have had for smoking cessation in the past?	3.5 (5.0)	6.0 (3.1)	8.5 (2.4)
9. To what extent were you able to learn new information that was based on your own personal needs?	4.5 (6.4)	5.8 (3.7)	7.0 (3.5)
10. To what extent were you concerned about electronic information (for example, privacy issues, sending information over the internet, etc.) as part of treatment? ^a^	10.0 (0.0)	10.0 (0.0)	8.3 (2.4)
11. To what extent did you want more person-to-person help as part of this treatment? ^a^	7.5 (3.5)	5.2 (4.2)	3.2 (4.7)
12. How helpful was the treatment in achieving your goals?	3.5 (5.0)	6.2 (3.6)	7.25 (3.2)
13. How easy to use was this treatment?	8.5 (.7)	7.5 (3.9)	7.5 (5)
14. How flexible was the treatment in terms of when you could access it?	8.0 (1.4)	8.2 (2.4)	10 (0.0)
15. Did the treatment help you learn and practice new skills in risky situations?	3.5 (5.0)	4.3 (4.8)	5.8 (5.1)
16. How likely is it that you would recommend this treatment to a friend?	5.0 (7.1)	7.7 (3.9)	10 (0.0)

**^a^** Reverse scored (10 is best).
